# Incorrect statistical method in parallel-groups RCT led to unsubstantiated conclusions

**DOI:** 10.1186/s12944-016-0242-3

**Published:** 2016-04-15

**Authors:** David B. Allison, Lisa H. Antoine, Brandon J. George

**Affiliations:** Nutrition Obesity Research Center and Department of Biostatistics, University of Alabama at Birmingham, Ryals Public Health Building, Room 140J, Birmingham, AL 35294 USA; School of Engineering, University of Alabama at Birmingham, Birmingham, AL 35294 USA; Department of Biostatistics, University of Alabama at Birmingham, Birmingham, AL 35294 USA; School of Public Health, University of Alabama at Birmingham, Ryals Public Health Building, Room 140J, Birmingham, AL 35294 USA

**Keywords:** Statistical analyses, Vegetable, Fruit, Cholesterol, Nutrition

## Abstract

The article by Aiso et al. titled “Compared with the intake of commercial vegetable juice, the intake of fresh fruit and komatsuna (*Brassica rapa* L. var *perviridis*) juice mixture reduces serum cholesterol in middle-aged men: a randomized controlled pilot study” does not meet the expected standards of *Lipids in Health and Disease*. Although the article concludes that there are some significant benefits to their komatsuna juice mixture, these claims are not supported by the statistical analyses used. An incorrect procedure was used to compare the differences in two treatment groups over time, and a large number of outcomes were tested without correction; both issues are known to produce high rates of false positives, making the conclusions of the study unjustified. The study also fails to follow published journal standards regarding clinical trial registration and reporting.

## Background

The conduct of rigorous randomized controlled trials (RCTs) is essential for progress in nutrition-related research [1]. In particular, rigorous tests of the causal effects of fruit and vegetable consumption on aspects of health would be valuable [2]. We therefore read with interest the paper by Aiso et al. [3] reporting results of an RCT of the effects of consumption of a commercial vegetable juice to that of the intake of fresh fruit and komatsuna (*Brassica rapa L. var. perviridis*) juice on serum cholesterol in men. Unfortunately, upon reading it became clear that incorrect statistical analyses were used, that the conclusions drawn in the paper are not supported by the analyses reported, and that there is insufficient adherence to RCT reporting guidelines [4], making it further difficult to determine the appropriateness of the analyses and the extent to which they adhere to original analytic plans.

### What the authors conclude

The authors conclude “Compared with the intake of commercial vegetable juice, the intake of fresh fruit and *B. rapa* juice is highly effective in reducing serum cholesterol.” As we will show below, this conclusion is not supported by the data and analyses presented.

### Why the analysis is incorrect

The stated goal of this study was to compare the effects of the two types of juices on anthropometric data, blood constituents, and dietary intake. To do so, the authors performed paired tests (baseline versus after 4 weeks) within each treatment group, and declared a significant difference between the juices when one juice’s test came up significant and the other juice’s test did not. This analysis strategy is frequently used in published literature, but is not statistically valid and can result in a type-1 error rate as high as 50% in trials with two groups [5]. As Allison et al. [6] wrote, given a parallel-groups RCT with measures of a continuous outcome at baseline and at endpoint; there are at least four legitimate ways to formally test the difference between two groups: (a) ignore the baseline data and analyze the endpoint data only with a simple independent samples t-test; (b) use a repeated measures ANOVA with one between-groups factor (treatment assignment) and one within-groups factor (time) and test the group-by-time interaction (*Y*_*ij*_ = *β*_0_ + *β*_1_*Treatment*_*i*_ + *β*_2_*Time*_*j*_ + *β*_3_*Treatment*_*i*_*Time*_*j*_ + *e*_*ij*_ *for i* = 1, …, *N*, *j* = 0, 1, *and* {*e*_*ij*_} *has a multivariate normal distribution*) [7, 8]; (c) analyze change scores (i.e., endpoint measurement minus baseline measurement) with a simple independent samples t-test; or (d) analyze the final outcome as an ANCOVA with one between-groups factor (treatment assignment) and one covariate (baseline scores) [9]. More details on these methods can be found in many classic experimental design books [7, 9, 10] and tutorial papers [6, 8, 10, 11]. Of note, method (d) (ANCOVA) is typically more powerful than method (c) (t-test on change scores) as it uses the observed pre-post correlation to more efficiently reduce the residual variance [11–13].

### Why the conclusions of the paper are not supported

Because a proper test *between groups* was not reported, we emailed the corresponding author of the paper, explained the statistical concern, and requested the standard deviation for the change in LDL-cholesterol and change in total cholesterol in each group or that they make the raw data available thereby allowing us to calculate the values ourselves. Unfortunately, we received no reply to our request. The ICMJE guidelines (http://www.icmje.org/icmje-recommendations.pdf) state “authors have a responsibility to respond appropriately and cooperate with any requests from the journal for data or additional information should questions about the paper arise after publication.” Given this, we suggest that Aiso et al. make the raw data from this trial available so that others may verify the results.

Although appropriate between-groups tests of the effects of treatment assignment on the key outcome variables were not reported, it seems unlikely that many of such tests could be significant. For total and LDL cholesterol on which Aiso et al’s conclusion claim is based, Aiso et al. do report the means and standard deviations for each variable within each of the treatment and control groups both at baseline and at endpoint. Using this information, we can implement choice *a* above^1^. If we do this for total cholesterol, the two-tailed *p*-value is 0.9480 (*t* = 0.0663; df = 14). If we do this for LDL cholesterol, the two-tailed *p*-value is 0.5525 (*t* = 0.6087; df = 14). In neither case is the result even close to significant, meaning that by this legitimate test, the appropriate conclusion would have been that there was no compelling evidence of a treatment effect.

Admittedly, the t-test only on endpoints is a relatively low power test; choice *c* above (a t-test on change scores) will usually be more powerful. Although it is clear that such a t-test would not be significant for LDL cholesterol (the groups had identical 9 mg/dl reductions), it is conceivable that the difference between the two groups in change of total cholesterol is statistically significant but we lack necessary information (such as the standard deviation of the change score) to conduct such a test. If Aiso et al. can show a statistically significant between-groups difference in the outcome variable, then their conclusion would be supported, but at present it is unsupported.

There is a concern regarding Aiso et al.’s reporting of *p*-values from 58 variables per treatment group (116 tests overall). Such a high number of tests would strongly suggest the use of a multiple testing correction to control the type-1 error rate [14] as one may expect approximately 5.8 significant findings to occur by chance alone if one tests 116 independent tests with a significance level of 0.05 and all the null hypotheses are true (i.e., there is really nothing to find). The smallest reported *p*-value was 0.012, far larger than what would be needed for significance under a Bonferroni (0.000431) or Sidak [15] (0.000442) correction. Although correlation between the 58 variables may reduce the extent of Type I error inflation and methods exist for correcting multiple correlated outcomes [16], those methods were not used in this article and without knowing the correlation between each variable it is impossible to quantify the extent of the inflation. Taken as a whole, it is plausible that many of the *p*-values reported as significant represent type-1 errors.

### Lack of trial registration

Articles published in *Lipids in Health and Disease* require adherence to BioMed Central’s editorial policies, http://www.lipidworld.com/about. BioMed Central follows the International Committee of Medical Journal Editors (ICMJE) guidelines, which necessitate clinical trials registration for RCT reports submitted to its journals. ICMJE defines a clinical trial as, “any research study that prospectively assigns human participants or groups of humans to one or more health-related interventions to evaluate the effects on health outcomes” [17]*.* ICMJE recommends that authors include the trial registration number in the abstract of the manuscript. This journal article does not include the clinical trials registration number. We emailed the authors to inquire about public clinical trials registry for this article, but received no response. Given the above, we believe that the authors should provide documentation of clinical trial registration.

## Conclusions

Clinicians, scientists, regulators, and the general public require and have a right to expect scientific evidence based on valid procedures [18] and free from spin [19] on which they can base decisions. The Committee on Publication Ethics [20] states that “Journal editors should consider retracting a publication if…they have clear evidence that the findings are unreliable [including]… as a result of …miscalculation or experimental error.” We believe that the conclusions of Aiso et al. [3] are unreliable as a result of using an incorrect statistical procedure.

## Abbreviation

RCT: Randomized controlled trial.

## Competing interests

The authors report no financial connection to the content of the paper discussed. David B. Allison and/or his institution have accepted funds from food companies, but not ones who, to his knowledge, market products discussed in this research.

## Authors’ contributions

David B. Allison conceived the paper. All three authors drafted sections of the manuscript and edited the entire paper. All authors read and approved the final manuscript.

## Author details

^1^Nutrition Obesity Research Center and Department of Biostatistics, University of Alabama at Birmingham, Ryals Public Health Building, Room 140J, Birmingham, AL 35294, USA. ^2^School of Engineering, University of Alabama at Birmingham, Birmingham, AL 35294, USA. ^3^Department of Biostatistics, University of Alabama at Birmingham, Birmingham, AL 35294, USA. ^4^School of Public Health, University of Alabama at Birmingham, Ryals Public Health Building, Room 140J, Birmingham, AL 35294, USA.

## Authors’ information

All authors are affiliated with the University of Alabama at Birmingham. David B. Allison is Associate Dean for Science in the School of Public Health, Distinguished Professor of Biostatistics, and Director of the NIH-funded Nutrition Obesity Research Center. Lisa H. Antoine is a doctoral student in Interdisciplinary Engineering. Brandon J. George is a statistician and holds a PhD in Biostatistics.

## Acknowledgements

Supported in part by NIH grants P30DK056336, R25DK099080, and R25HL124208. The content is solely the responsibility of the authors and does not necessarily represent the official views of the National Institutes of Health or any other organization.

## Footnotes

1 We conducted our calculations with the free public software at this site http://www.graphpad.com/quickcalcs/ttest2/ so that anyone could reproduce our calculations.
