# Supplementary material for: Incorrect statistical method in parallel-groups RCT led to unsubstantiated conclusions
Source: Lipids Health Dis. 2016 Apr 15;15:77. doi: 10.1186/s12944-016-0242-3 (PMC4869669; doi:10.1186/s12944-016-0242-3)
Supplement: Related texts

# Reply: Response to “Incorrect statistical analyses led to unsubstantiated conclusions”

**Authors:** By Izumi Aiso, Hiroko Inoue, Yukiko Seiyama, Toshiko Kuwano

**Affiliations:** Shizuoka Prefectural Chubu Public Health Center, ᅟ, ᅟ; Department of Nutrition and Health Sciences, Faculty of Food and Nutritional Sciences, Toyo University, ᅟ, ᅟ; Department of Food and Nutritional Sciences and Environmental Health Sciences, Graduate School of Integrated Pharmaceutical and Nutritional Sciences, University of Shizuoka, 52-1 Yada, Suruga-ku, Shizuoka 422-8526 Japan

## Introduction

Here we respond to the commentary on our article by Allison et al. As an overview of the issues they raise, we have selected the following statement from their commentary.

“Although the article concludes that there are some significant benefits to their komatsuna juice mixture, these claims are not supported by the statistical analyses used. An incorrect procedure was used to compare the differences in two treatment groups over time such that no direct between-group comparison was done, and a large number of outcomes were tested without correction.”

At various points their commentary, they raise questions about the following aspects of our study: 1) statistical analysis, 2) dietary survey, and 3) clinical trial registration. Below, we respond to each of these questions in turn.

## Statistical analysis

Before we turn to the detailed statistical issues that Allison et al. raise in their commentary, we would first like to point out that, when our article was reviewed by the journal’s referees, both referees specifically stated that our statistical analysis did not need further verification. Referee 1 wrote in his/her review:

“**Statistical review:** No, the manuscript does not need to be seen by a statistician.”

And Referee 2 wrote in his/her review:

“**Statistical review:** No, the manuscript does not need to be seen by a statistician.”

As the referees thought that a statistical review was unnecessary, we felt confident that we had used the appropriate statistical methods. Therefore, we were surprised that our methods were criticized by Allison et al. However, we were also very interested in their interpretation, so we followed their suggested method for analyzing our data.

In their commentary, Allison et al. state:

“The stated goal of this study was to compare the effects of the two types of juices on anthropometric data, blood constituents, and dietary intake. To do so, the authors performed paired tests (baseline versus after 4 weeks) within each treatment group, and declared a significant difference between the juices when one juice's test came up statistically significant (defined by the authors as p<0.05) and the other juice's test did not. This analysis strategy of comparing nominal significance of within group changes (done with either paired-sample parametric or non-parametric tests) is frequently used in published literature, but is not statistically valid and can result in a false positive rate as high as 50% in trials with two groups of equal size.”

and

“there are several legitimate ways to formally test the difference between two groups: (a), (b), (c).”

[…]

“Method (c) (ANCOVA) is typically more powerful than method (b) (t-test on change scores). As it uses the observed pre-post correlation to more efficiently reduce the residual variance.”

We thank Allison et al. for pointing out this new statistical approach. Like the other researchers in the field, we were not aware of this more advanced method when we wrote our paper. However, we were pleased to be able to apply it to the analysis of our data following the suggestion of Allison et al. We return to that analysis below, but first we would like to clarify some issues related to the Wilcoxon signed rank test that we used in our study.

The goal of this study was to examine changes in various parameters in the intervention group and the control group before and after their respective juice interventions. In analyzing our data, we performed both a paired t-test and a Wilcoxon signed rank test. Both tests showed that the concentration of total cholesterol and LDL-cholesterol in the intervention group were significantly lower after 4 weeks compared with the baseline values. However, we chose to report the results of the Wilcoxon signed rank test, as that test is more appropriate for small sample sizes that do not have normal distributions. In retrospect, we now think that it would have been clearer to include the reason that we selected the Wilcoxon test in our article. We apologize for any misunderstanding that this omission may have caused.

As Allison et al. raised questions about our statistical methods, we first rechecked the results of our original statistical analysis. After that, we applied the statistical analysis that they have suggested.

**a. Re-application of Wilcoxon signed rank test.** When we repeated the Wilcoxon signed rank test to ensure that the results of our original test were accurate, we found that the results were the same as those we had originally published.

**b. Application of analysis of covariance (ANCOVA).** In their commentary, Allison et al. state:

“there are several legitimate ways to formally test the difference between two groups: (a), (b), (c).”

[…]

“Method (c) (ANCOVA) is typically more powerful than method (b) (t-test on change scores). As it uses the observed pre-post correlation to more efficiently reduce the residual variance.”

Following this suggestion, we re-examined our data using ANCOVA. Data were analyzed using SPSS for Windows version 15.0 J computer software. We conducted the ANCOVA test by adjusting for levels of age, BMI, and each variable. The results did not show a significant difference for the parameters in either group. We speculate that this may be due to the small number of subjects participating in the study. In the light of this new analysis, we think that the conclusions stated in our original article should be moderated. We return to this point in our conclusion below.

## Dietary survey

In their commentary, Allison et al. state:

“There is also a concern regarding Aiso et al.’s reporting of *p*-values from 58 variables per treatment group (116 tests overall).”

We would like to clarify the nature of the dietary survey used in our study. We used the “brief-type self-administered diet history questionnaire” (BDHQ), which is a standard dietary history survey instrument used in Japan [21, 22]. The BDHQ is used to calculate intake values such as energy and nutrients based on information about 58 types of food and drink. The questionnaires are batch processed at the Diet History Questionnaire Support Center. Because the Center calculates nutrient values from the intake frequency of the 58 food and drink items and then sends those values to us, we cannot individually manipulate the food and drink items as variables afterwards by ourselves. We can only perform our analysis based on the data categories provided by the Center, and therefore we cannot perform a more detailed statistical analysis on the 58 items.

## Clinical trial registration

In their commentary, Allison et al. state:

“The study also fails to follow published reporting guidelines for this journal and the scientific community overall regarding clinical trial registration and reporting.”

We have registered our study with the UMIN-CTR Clinical Trial database. The information can be found at: https://upload.umin.ac.jp/cgi-open-bin/ctr/ctr.cgi?function=brows&action=brows&recptno=R000022765&type=summary&language=E.

## Conclusion

The statistical test that we used in our article was considered appropriate by both of the journal’s reviewers. We have rechecked the results of that test, and have obtained the same result. In addition, we have carried out the ANCOVA test suggested by Allison et al. ANCOVA did not show a significant difference between the intervention group and the control group. We thank Allison et al. for providing us with this insight. In future studies we plan to increase the number of subjects and reinvestigate the effect. We will also consider conducting a crossover study similar to that of Lee et al. in the future [23].

Based on the insights that we have gained from the suggestions of Allison et al., we think that the conclusion originally drawn in our article should be moderated to be stated as follows:

**Compared with the intake of commercial vegetable juice, the intake of fresh fruit and*B. rapa*juice may be effective in reducing serum cholesterol**.

We would like to once again thank Dr. Allison and colleagues for their thoughts on our article.
